# Persistent Fever in Bilateral Septic Cavernous Sinus Thrombosis: A Case Report

**DOI:** 10.7759/cureus.69800

**Published:** 2024-09-20

**Authors:** Ryan Clydesdale, Katelyn Stoker, Susan Bannon

**Affiliations:** 1 Department of Internal Medicine, Western Michigan University Homer Stryker M.D. School of Medicine, Kalamazoo, USA; 2 Department of Internal Medicine Pediatrics, Western Michigan University Homer Stryker M.D. School of Medicine, Kalamazoo, USA

**Keywords:** mrsa bacteremia, otolaryngological surgery, septic cavernous sinus thrombosis, source control, sphenoidal sinusitis

## Abstract

Septic cavernous sinus thrombosis (SCST) is a rare and life-threatening condition characterized by thrombus formation within the cavernous sinus, typically resulting from the spread of infection from facial, paranasal sinus, or dental origins. We report the case of a middle-aged man with a history of methicillin-resistant Staphylococcus aureus (MRSA) skin infections who presented with severe neck pain, fever, and bilateral eye swelling. The patient was bacteremic with MRSA and found on imaging to have thickening of extraocular muscles and air-fluid levels present within the sphenoid sinuses. Despite aggressive antibiotic and anticoagulant therapy, the patient’s condition deteriorated. Imaging showed extensive intracranial thromboses involving the cavernous sinuses. Given the lack of clinical improvement and persistent bacteremia, otolaryngological surgical intervention was undertaken. Post-surgery, the patient showed marked improvement, underscoring the critical role of source control in managing SCST. This case highlights the importance of considering surgical options in the treatment of SCST to prevent severe complications and improve patient outcomes.

## Introduction

Septic cavernous sinus thrombosis (SCST) is a rare and serious medical condition characterized by the formation of blood clots within the cavernous sinuses of the brain. The cavernous sinuses, located bilaterally on either side of the sella turcica, house multiple cranial nerves as well as a complex network of veins that drain blood from the face, eyes, and cerebral hemispheres [1]. This condition typically arises from the spread of infectious agents, such as bacteria, originating from paranasal sinus or facial infections. Ophthalmologic complications are a hallmark of SCST, with orbital venous congestion potentially resulting in proptosis, chemosis, and ophthalmoplegia. The proximity of the cavernous sinuses to vital cranial nerves such as the oculomotor and abducens nerve predisposes patients to cranial nerve palsies and functional vision loss. With an annual diagnosis rate of 4.5 per 1,000,000 individuals, each case is important to highlight to further expand the limited existing medical literature on this condition [2]. Standardized treatment guidelines are sparse given the rarity of the condition, necessitating the collection of more data from which such treatment standards may be formed. 

## Case presentation

**Figure 1:**
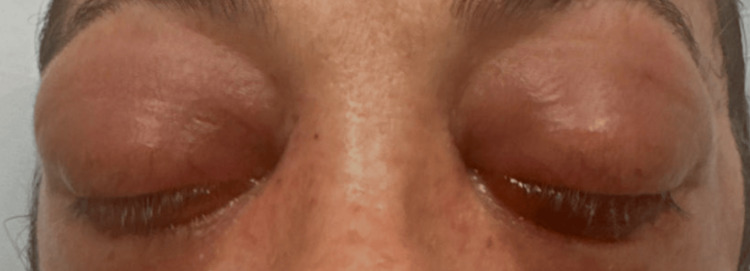
Bilateral proptosis on admission

**Figure 2:**
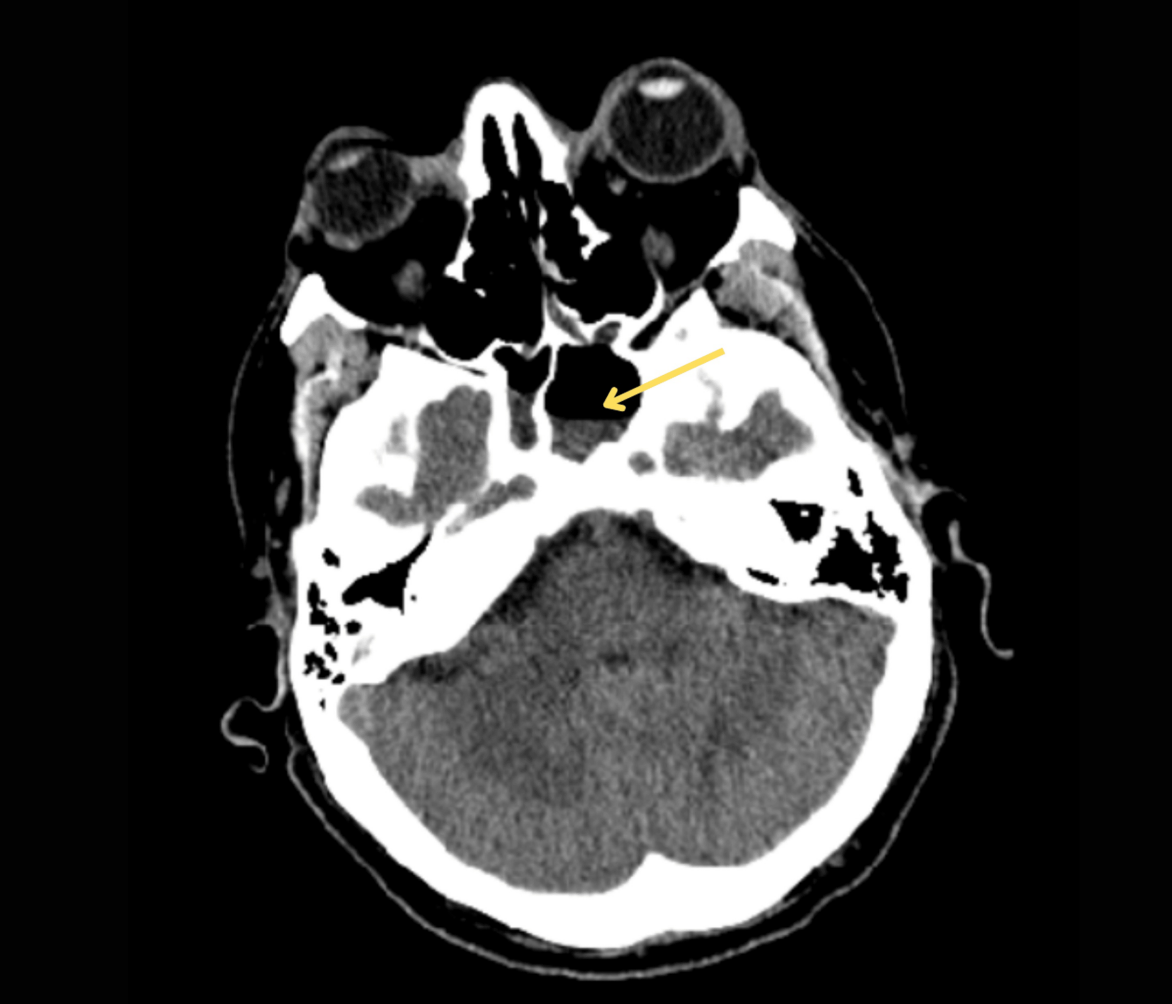
CT brain showing air-fluid levels present within the sphenoid sinuses bilterally

**Figure 3:**
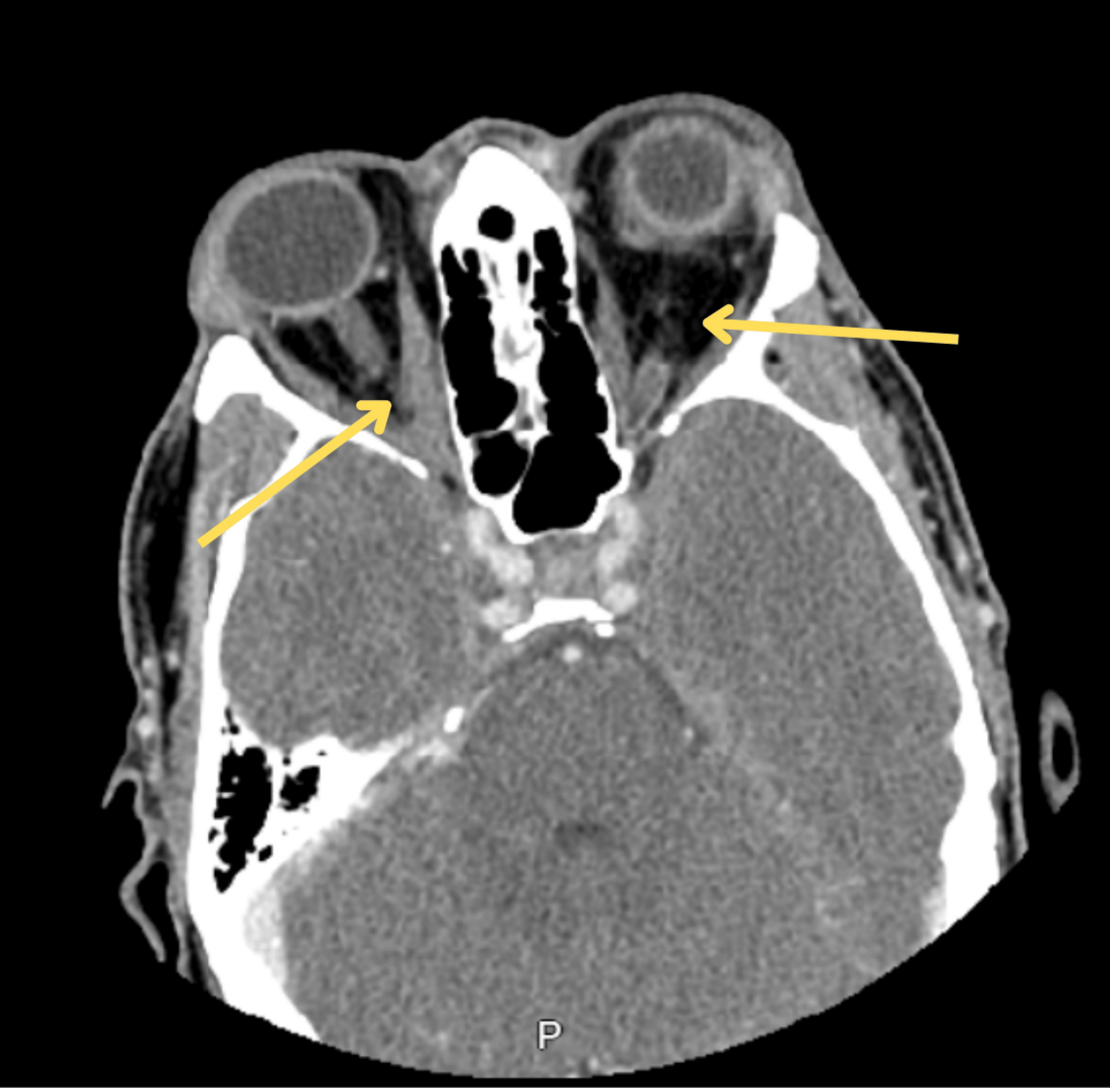
CT orbit showed bilateral proptosis with slight thickening of the muscle bellies of the superior, medial, and inferior rectus musculature with adjacent fat stranding

A 39-year-old immunocompetent man presented to the emergency department after five days of worsening right-sided neck pain and stiffness, fever, photophobia, and one day of bilateral eye swelling. He reported a remote history of methicillin-resistant Staphylococcus aureus (MRSA) skin infection with the recent development of multiple furuncles three weeks before presentation. He reported rupturing these furuncles, including one above his upper lip and one on the right knee. The rupture of the furuncle on the knee led to severe right knee pain, swelling, and redness, which had resolved before presentation. He denied recent or past IV drug use, other known wounds, or recent dental procedures. He also denied any recent illnesses, sinus congestion, runny nose, sore throat, cough, or earache. The physical examination was notable for drenching diaphoresis, neck rigidity, and neck tenderness. Examination of the eyes revealed left ophthalmoplegia with a fixed and dilated pupil, photophobia, chemosis, and significant bilateral proptosis with periorbital edema (Figure 1). Initial workup revealed leukocytosis, lactic acidosis, mild hyponatremia, hypochloremia, and mild metabolic acidosis (Table 1). CT brain without contrast showed distended/dilated veins within left and right supraorbital fat and air-fluid levels present within the sphenoid sinuses (Figure 2). CT orbit with contrast revealed bilateral proptosis with slight thickening of the muscle bellies of the superior medial and inferior rectus muscles with fat stranding (Figure 3). The patient was started on therapeutic heparin, IV vancomycin, IV ceftriaxone, and dexamethasone 10 mg q6 hr for suspected meningitis and concern for intracranial thrombus. Blood cultures collected on presentation grew MRSA with time to the first positive result of less than nine hours. Lumbar puncture was not pursued at this time due to the patient's bacteremia likely sharing the same etiology as the meningitis. 

**Table 1:** Admission laboratory values WBC: white blood cells; TSH: thyroid stimulating hormone

Laboratory Finding	Admission	Reference
WBC, x10^9^ cells/L	21.8	4.5-11.0
Lactic acid, mmol/L	2.7	0.5-2.2
Procalcitonin, ng/mL	2.48	< 0.10
Sodium, mEq/L	133	135-145
Chloride, mEq/L	97	98-108
CO2, mEq/L	17	23-32
TSH, u[iU]/mL	0.48	0.27-4.20

**Figure 4:**
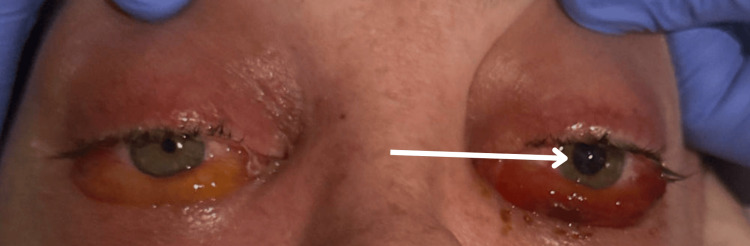
Appearance of eyes on hospital day two with left pupillary defect and conjunctival injection

**Figure 5:**
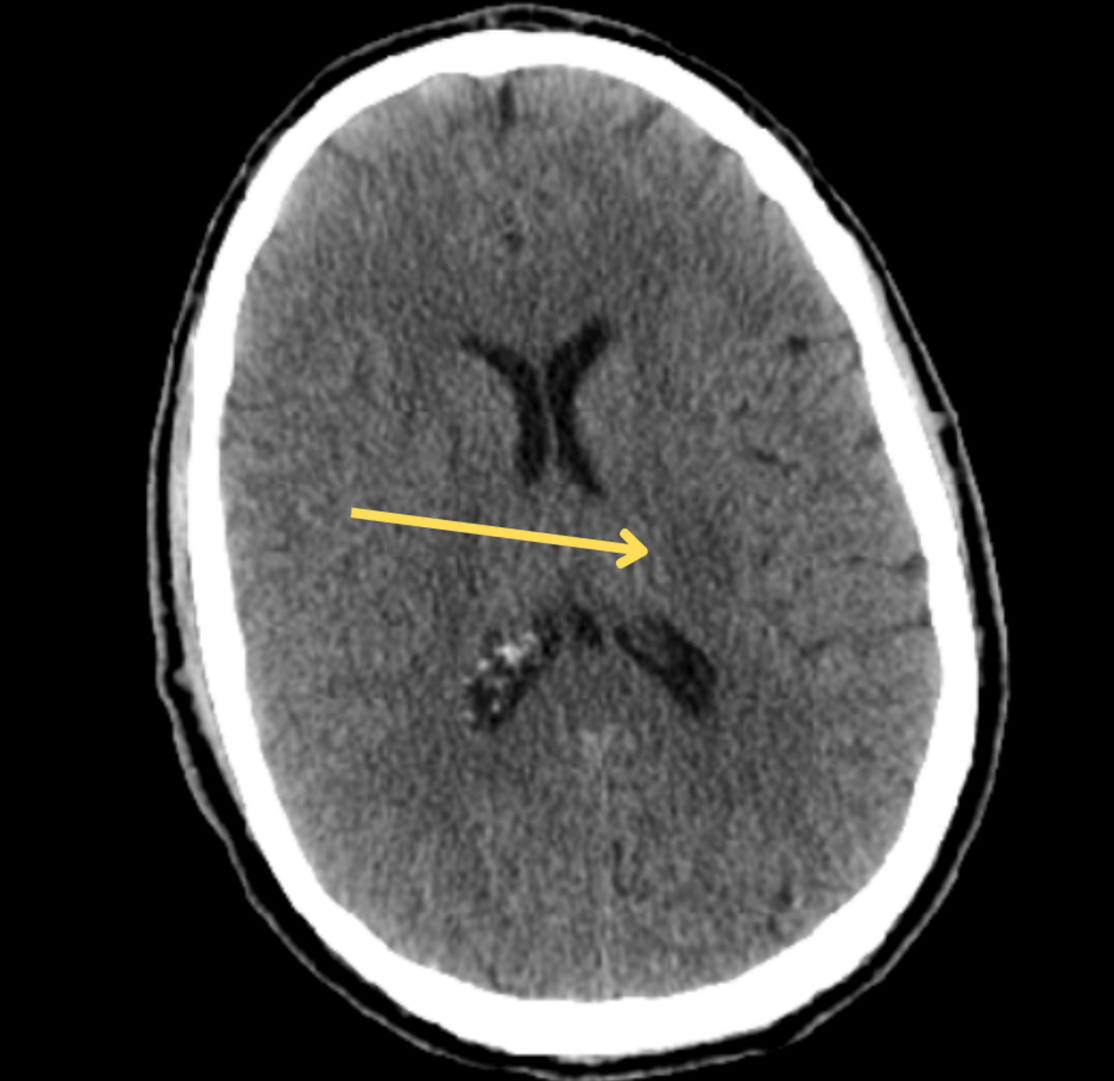
CT head showing a small area of new low attenuation in the left temporal lobe adjacent to the ventricle, raising suspicion for acute infarction

**Figure 6:**
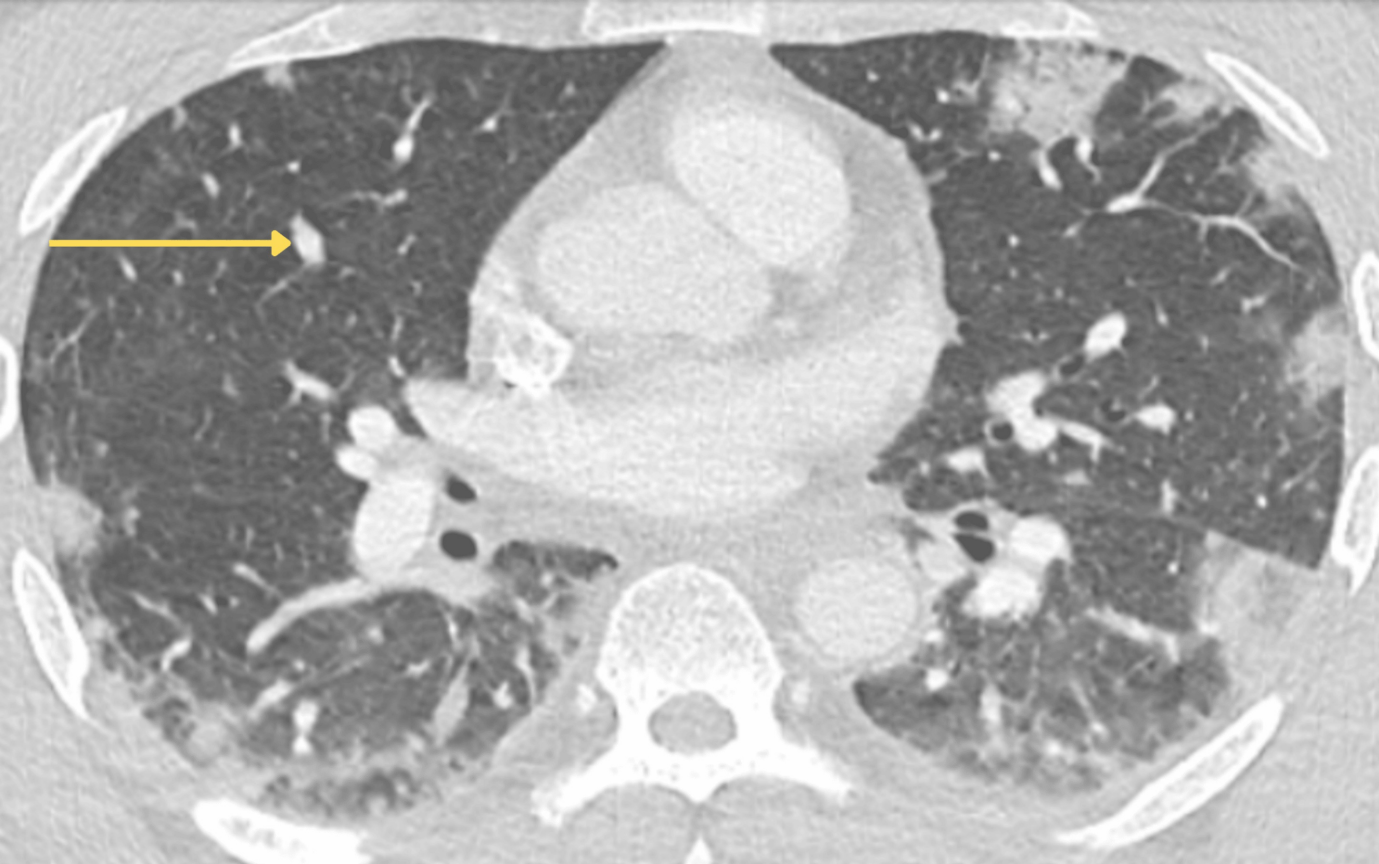
CTA chest on hospital day two showed multiple ill-defined peripheral nodules concerning septic emboli and bilateral ground glass opacities CTA: computed tomography angiography

On hospital day one, an MRI brain showed mild increased signal intensity on diffusion-weighted sequence in the cavernous sinus greater on the right and mildly prominent intermediate signal intensity on T2-weighted sequence within the cavernous sinus bilaterally, which were concerning for cavernous sinus thrombosis. Air-fluid level in the sphenoid sinus was concerning for infection in the sinus. On hospital day two, the patient began to develop new neurological deficits consisting of slurred speech, right facial droop, and right-hand weakness, which were accompanied by worsening posterior neck pain beginning at the occiput and progressing down into the neck. The patients left ophthalmoplegia with a fixed and dilated pupil and remained unchanged from admission (Figure 4). In addition, he reported new chest pain and was noted to have worsening tachypnea, tachycardia, and hypoxia. CT head at that time revealed a small area of new low attenuation in the left temporal lobe adjacent to the ventricle, raising suspicion of acute infarction (Figure 5). CT chest with contrast revealed multiple ill-defined peripheral nodules concerning septic emboli and bilateral ground glass opacities, but no evidence of pulmonary embolism (Figure 6). The transthoracic echocardiogram was negative for valvular vegetation. Otolaryngology was consulted but declined intervention at that time. Antibiotic treatment was adjusted to broaden coverage with the addition of metronidazole and the substitution of cefepime for ceftriaxone. The patient began requiring increasingly large doses of heparin to remain anticoagulated. Due to pharmacy concerns of heparin resistance, the patient’s anticoagulant was changed to argatroban.

**Figure 7:**
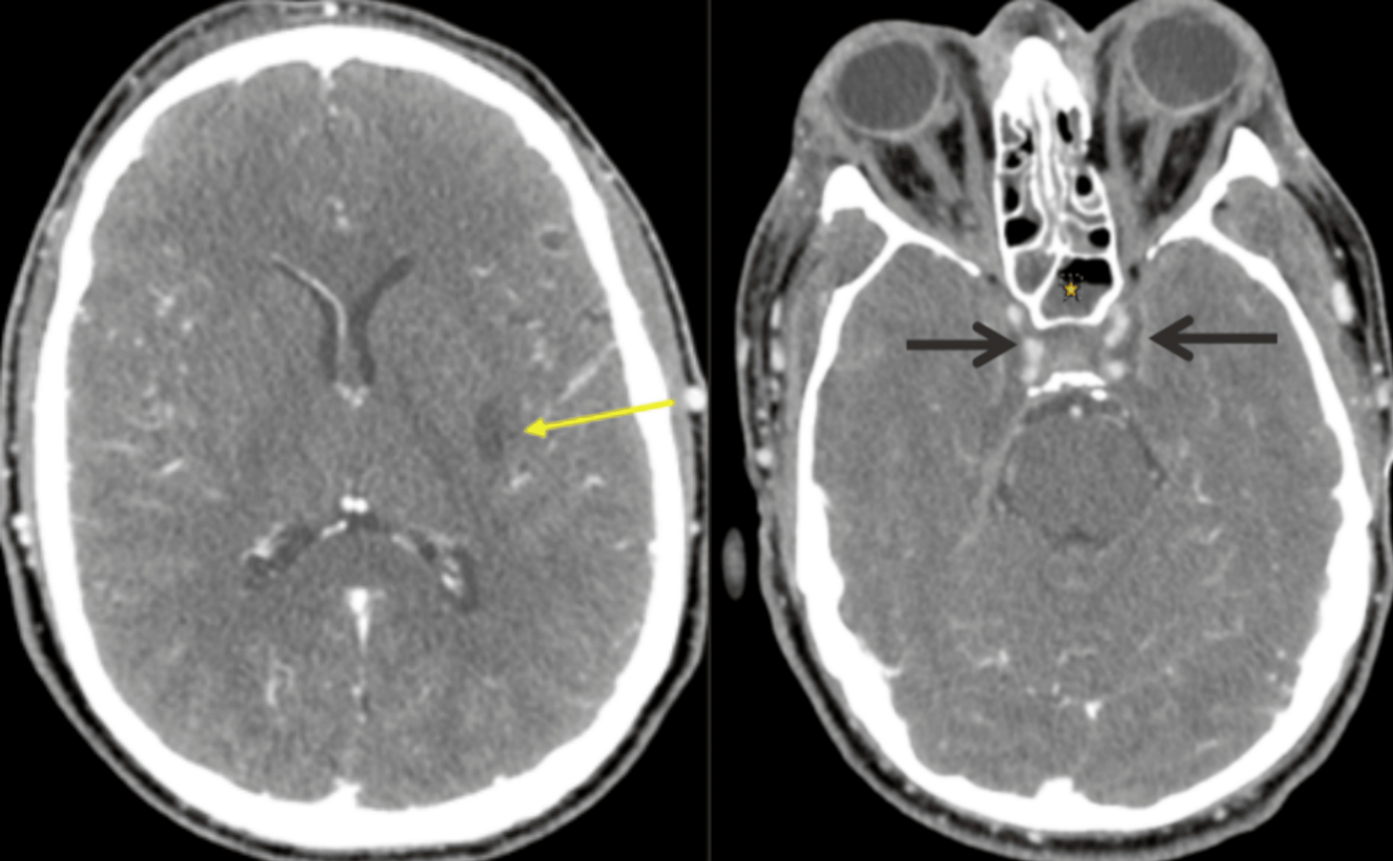
CT venogram of the head (day five) left: infarct in the left lentiform nucleus; right: filling defect in bilateral cavernous sinuses (arrows) consistent with thrombosis, and air-fluid levels in the sphenoid sinuses (star)

The patient showed minimal improvement despite aggressive antibiotic treatment and continued to develop daily fevers. On physical exam, the patient’s bilateral proptosis remained significant, with the patient complaining of pain in his face, orbits, and neck. The patient’s neurological exam continued to worsen with increased right-sided weakness and declining mental status. Due to a lack of improvement and persistent bacteremia, further imaging was pursued on hospital day five. A CT venogram of the head again showed areas of infarct in the left lentiform nucleus and medial left temporal lobe, a new small mycotic aneurysm in the M2 branch of the middle cerebral artery, a peripherally enhancing right C1 joint effusion, linear nonocclusive thrombi in the bilateral sagittal sinuses and proximal internal jugular veins, thrombosis of bilateral superior ophthalmic veins and right facial vein, and complete opacification of the sphenoid sinuses with air-fluid levels (Figure 7). The spread of infection and thrombosis was speculated to have occurred in a posterior-to-anterior direction, potentially arising from the C1 joint effusion, extending to the cavernous sinuses, then to the superior ophthalmic veins and right facial vein. On hospital day six, linezolid and pyridoxine were added to vancomycin per the recommendation of infectious diseases specialists. 

**Figure 8:**
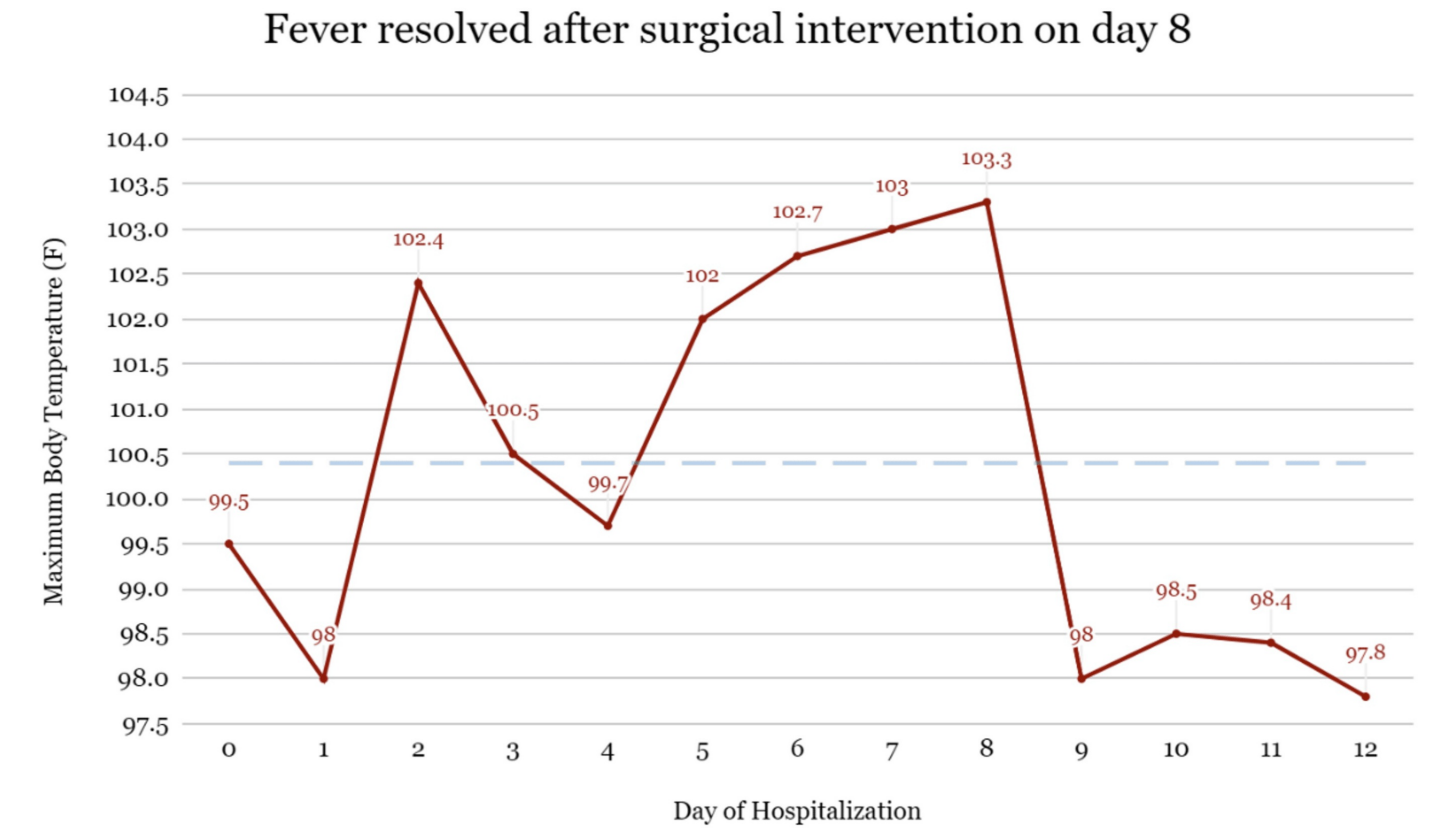
As reflected in the graph above, this patient continued to have daily fevers (as well as new septic emboli) despite one week of proper antibiotic treatment, though bacteremia resolved by day five

Given the patient’s poor prognosis and stagnant clinical course, the consulting otolaryngologist proceeded with bilateral maxillary, antrostomy, total ethmoidectomy and sphenoidotomy, and frontal sinusotomy and septoplasty on hospital day eight for source control. Intraoperatively, the patient was found to have bilateral severe inflammation in the sphenoid sinuses with less noticeable inflammation in the remainder of the sinuses. Paranasal sinus cultures taken during surgery were positive for MRSA. On postoperative day one, there was the resolution of fever, and a physical exam showed significant improvement in bilateral eye swelling (Figure 8). In the days following, there was a steady improvement in vital signs and leukocytosis. Follow-up transesophageal echocardiogram on hospital day 14 remained negative for vegetation. The patient was discharged to inpatient rehab on hospital day 21 in stable condition, though with permanent unilateral vision loss, ophthalmoplegia of the left eye, and persistent right-sided motor deficits. 

## Discussion

Septic cavernous sinus thrombosis (SCST) is a rare condition with high morbidity and mortality characterized by thrombus formation within the cavernous sinus resulting most often from contiguous spread of facial, paranasal sinus, or dental infections. In the pre-antibiotic era, this condition had a 100% fatality rate [3]. Mortality rates have been reported as ranging from 11% to 30% in the antibiotic era across various studies [4,5]. Despite modern antibiotic availability, SCST often leads to severe complications, as in this case, such as permanent neurological deficits, due to its proximity to critical structures such as cranial nerves and the internal carotid artery [1]. 

SCST typically occurs due to the spread of infection from the face or paranasal sinuses [1]. The dural venous sinuses lack valves, allowing bidirectional blood flow that enables infectious agents to spread to various parts of the brain from the face via the cavernous sinuses [6]. Skin infections in the "danger triangle" of the face, which extends from the corners of the mouth to the bridge of the nose, pose a significant risk due to the unique venous connections to the cavernous sinuses. This area is a well-established anatomical region of heightened concern, with a study from 1937 finding many cases of cavernous sinus thrombosis resulting from furuncles on the upper part of the face [7]. This patient's history of manipulating a furuncle above his upper lip likely facilitated the spread of MRSA, culminating in severe sphenoid sinusitis, cavernous venous thrombosis, and subsequent neurological complications. It is important to consider that the infection may have originated from the knee lesion rather than the facial furuncle. The patient’s history of knee pain, swelling, and systemic symptoms following the rupture of the knee lesion raises the possibility of indolent bacteremia, leading to infection at the C1 joint space. This could have subsequently spread posteriorly to anteriorly, resulting in thrombosis of the cavernous sinuses and associated veins. Although the direct link to sinusitis is unclear, both the knee and facial lesions should be considered as potential sources of infection in this case. 

Bacteria are the most common cause of SCST, with *Staphylococcus aureus* being responsible for about two-thirds of cases [1]. Other common pathogens include *Streptococcus spp. *(around 20% of cases),* pneumococcus* (5%), and gram-negative bacteria such as *Proteus, Hemophilus, Pseudomonas, Fusobacterium,* and *Bacteroides*, as well as gram-positive bacteria such as *Corynebacterium* and *Actinomyces* [1]. Initial empiric bacterial antibiotic coverage for this patient included an anti-staphylococcal agent with activity against MRSA (vancomycin), a third-generation cephalosporin (ceftriaxone), and anaerobic coverage (metronidazole), all of which are sufficient for coverage of what was subsequently identified as MRSA bacteremia [1]. Anti-fungal treatment was considered but not pursued due to a lack of fungal infection risk factors and no evidence of fungal organisms on blood cultures. Despite aggressive antibiotic and anticoagulant therapy, the patient continued to exhibit persistent fevers and worsening neurological deficits. These symptoms, along with imaging findings of progressive spread of thrombosis, necessitated a reassessment of the treatment strategy. 

Surgical intervention is a documented option for the treatment of septic cavernous sinus thrombosis. A 2016 literature review of cases of SCST occurring between January 1980 and July 2015 showed that surgery was performed in 54% of 88 total cases [4]. Most of these procedures focused on the paranasal sinuses to target the source of infection (e.g., ethmoidectomy, sphenoidotomy, maxillary antrostomy), which was the focus of surgery in this patient as well [4]. Other documented interventions included incision and drainage of abscesses, dental extractions, craniotomy to evacuate subdural empyema, and orbital decompression [4]. Additionally, no specific surgical interventions are recommended for the cavernous sinuses themselves [1]. Although surgical intervention is a documented option, clinicians may be unfamiliar with the condition due to its rarity, complexity of presentation, or variability of infectious source and thus feel hesitant to operate on such acutely ill patients. Further research into the optimal timing and clinical criteria for surgical intervention is needed to guide clinical practice, leading to improved patient outcomes. 

This case highlights the importance of surgical intervention to attain source control in managing SCST. The paranasal sinus involvement was likely serving as a depot of infection for ongoing fever and bacteremia unresponsive to IV antibiotics. The patient demonstrated rapid improvement in fever, leukocytosis, and clinical instability only after the source of infection (severe sinusitis) was controlled with otolaryngological intervention. Given the clinical course leading up to surgical intervention, it is highly probable that the patient would have at the very least suffered significant morbidity, if not mortality, without source control.

## Conclusions

Though rare, septic cavernous sinus thrombosis is a serious condition with high morbidity and mortality even in the era of antibiotic availability. Early recognition and diagnosis of the condition are crucial for timely intervention. When aggressive medical therapy alone does not lead to rapid resolution of symptoms, surgical intervention should be considered early in the course to attain source control. A multidisciplinary approach including both aggressive medical and surgical treatments may help improve outcomes in patients with septic cavernous sinus thrombosis. Surgical intervention is documented in the clinical literature, but clinicians may feel hesitant due to the complexity and rarity of such cases. More research is needed regarding optimal timing and clinical criteria for surgical intervention to help inform standardized treatment guidelines moving forward.
